# Beyond the cortex, early deficits, late recovery: a systematic review of language outcomes and subcortical mapping after awake craniotomy for diffuse gliomas

**DOI:** 10.1186/s41016-026-00452-8

**Published:** 2026-11-09

**Authors:** Maher Nassor, Parsa Mousazadeh, Michaela Bramhall, Mohammed Sanaulla, Elizabeth Martini, Alex Yacoub, Oyeleye Egunjobi

**Affiliations:** 1 School of Medicine, University of Sunderland, Tyne and Wear, UK; 2 School of Medicine, University of Manchester, Manchester, UK; 3 School of Medicine, University of Buckingham, Buckingham, UK; 4 School of Medicine, University of Warwick, Warwick, UK; 5 School of Medicine, University of Cardiff, Cardiff, UK; 6 Department of Neurosurgery, University Hospitals Birmingham, Birmingham, UK; 7 Bowen University Teaching Hospital, Ogbomoso, Nigeria

**Keywords:** Awake craniotomy, Diffuse gliomas, Direct electrical stimulation, Eloquent brain areas

## Abstract

**Background:**

To evaluate immediate and long-term postoperative language outcomes in patients undergoing awake craniotomy with direct electrical stimulation (DES) for diffuse gliomas in eloquent regions.

**Methods:**

PRISMA-compliant systematic review (PROSPERO: CRD420261342690) of adults (≥ 18 years) with supratentorial diffuse glioma in the language-dominant hemisphere enrolled in both randomised and non-randomised studies of awake craniotomy with direct electrical stimulation and language mapping. Databases searched included PubMed, MEDLINE, Embase, and Cochrane CENTRAL (from 2010 to March 2026). Study selection, data extraction, and risk of bias assessment were performed independently by multiple reviewers using standardised tools (JBI Critical Appraisal Checklists). Due to methodological heterogeneity, narrative synthesis was performed.

**Results:**

Ten studies (532 patients enrolled; 55.3% male) published 2011–2025 were included; tumour grade was reported inconsistently, with higher-grade tumours predominating in the larger cohorts and at least 114 patients having WHO grade 4 disease. Early postoperative deficits ranged from 2.2 to 100% depending on assessment timing and measure (immediate post-op: up to 100%; day 7 to week 1: 2.2–22%). Persistent deficits at ≥ 3 months ranged from 0 to 47%. In three studies reporting whole-cohort outcomes with standardised assessment, rates were 0–5.6%. Papagno et al., using 3-month cut-offs, found that 47% of patients with uncinate fasciculus resection had impairment in famous face naming versus 10% with the tract preserved. The general trend was early decline followed by improvement, with most reported deficits resolving by around 3 months. Heterogeneous deficit definitions and inconsistent baseline assessment preclude treating this as a prognostic pattern, and resections involving the uncinate fasciculus were a notable outlier, underscoring the need for systematic subcortical tract mapping.

**Conclusion:**

Awake craniotomy with DES remains the gold standard for preserving language. Early deficits are common but predominantly transient, and reported deficit rates appear closely associated with the timing of assessment. Persistent deficits assessed at ≥ 3 months were low in most studies, although rates varied considerably with the outcome measure used and with subcortical tract involvement. Subcortical tract involvement warrants systematic mapping. We advocate for the global adoption of standardised perioperative linguistic protocols to improve data comparability.

**Systematic review registration:**

PROSPERO CRD420261342690

**Supplementary Information:**

The online version contains supplementary material available at https://doi.org/10.1186/s41016-026-00452-8.

## Background

Diffuse gliomas are primary brain tumours arising from neuroglial stem cells and are characterised by a highly infiltrative growth pattern that presents significant challenges for surgical resection [1, 2]. Diffuse gliomas are the most frequent malignant primary central nervous system tumours in adults, representing around 80% of the 12,000 new malignant primary brain tumours diagnosed annually in the UK [1, 3]. The 2021 World Health Organisation (WHO) classification system integrates molecular features in the classification of diffuse gliomas including Isocitrate Dehydrogenase (IDH) mutation along with 1p and 19q codeletion status. These molecular characteristics are associated with prognosis and treatment response, with IDH mutation and 1p/12q codeletion conferring a more favourable outcome than IDH-wildtype disease, highlighting the importance of careful preoperative assessment to guide surgical decision-making [1].

Given the highly infiltrative nature of diffuse gliomas and the influence of molecular features, surgical management prioritises maximising tumour resection while preserving neurological function. Extent of resection (EOR) is recognised as a critical modifiable factor in the surgical treatment of diffuse gliomas. Consistent evidence associates a greater EOR with an improvement in quality of life (QoL), progression-free survival and overall survival for patients with gliomas [4–6]. However, gross total resection (GTR) is not always achievable due to the highly infiltrative nature of diffuse gliomas, particularly in language-eloquent regions where preservation of neurological and language function must be balanced against maximal tumour resection. This challenge has driven the development of intraoperative techniques aimed at optimising tumour resection in eloquent areas.

One established intraoperative approach is an awake craniotomy with direct electrical stimulation (DES), which enables localisation of functional tissue in real time during glioma resection. The infiltrative growth pattern of gliomas often results in tumours located adjacent to functional brain regions, requiring intraoperative mapping using high- or low-frequency stimulation at cortical or subcortical boundaries to identify essential functional tissue [7]. Using DES, intraoperative stimulation mapping (ISM) facilitates identification of language-specific functional sites through patient responses to language tasks, where disruptions such as speech arrest indicate language-positive sites requiring preservation during resection. Other preoperative tools including fMRI, MEG, DTI, and nTMS lack the accuracy and specificity demonstrated by ISM, supporting the role of ISM as the gold standard for functional tissue mapping during surgical resections [7].

Despite advances in intraoperative functional mapping, postoperative language deficits remain common in the early postoperative period and vary substantially in both frequency and duration. In many cases, these early postoperative deficits are transient, resolving over time while others may persist permanently [8]. These permanent language deficits can include aphasia or speech disturbances, which can negatively affect a patient’s social and occupational functioning. However, heterogeneity in postoperative language assessment methods and follow-up strategies has resulted in inconsistent synthesis of postoperative language outcomes across the literature.

Given the heterogeneity in reported postoperative language outcomes, this systematic review aims to evaluate language outcomes following awake craniotomy with DES in adults with diffuse gliomas located in language-eloquent areas. It will place specific focus on early (< 3 months) and late (≥ 3 months) postoperative outcomes, extent of resection achieved, and the influence of language pathway involvement on postoperative language outcomes. This review aims to characterise the spectrum of language deficits reported and identify patterns of language recovery following surgery.

## Methods

Our systematic review was conducted in accordance with the Preferred Reporting Items for Systematic Reviews and Meta-Analyses (PRISMA) guidelines [9] and was prospectively registered on PROSPERO, registration number: CRD420261342690.

### Information sources and search strategy

A systematic search was performed across PubMed, MEDLINE via Ovid, Embase via Ovid, and Cochrane CENTRAL on 01/03/2026. The search was limited to human studies, English-language publications, and articles published from 2010 onward. The search strategy combined controlled vocabulary and free-text terms across four concept blocks joined with the AND operator: (i) diffuse glioma (including MeSH/Emtree glioma and neurosurgical procedure headings, low-grade glioma, diffuse astrocytoma, oligodendroglioma, IDH status, and WHO grade 2–3 descriptors); (ii) awake surgery and intraoperative stimulation mapping (awake craniotomy, awake surgery, intraoperative mapping, cortical stimulation, direct electrical stimulation); (iii) comparator or adjunctive intraoperative technique (asleep surgery, general anaesthesia, neurophysiological and intraoperative monitoring, motor and somatosensory evoked potentials, diffusion tensor imaging, tractography, functional mapping); and (iv) language outcome (language, aphasia, speech, naming, fluency, comprehension, dominant or left hemisphere, Broca, Wernicke, eloquent area, or cortex). A NOT block excluded high-grade descriptors and animal studies. Additionally, we conducted forward and backward citation searches by examining the reference lists of included articles, relevant reviews, and studies that cited the included research.

### Eligibility criteria

The eligible population comprised adults aged 18 years or older with supratentorial diffuse glioma located in the language-dominant hemisphere, involving the language-eloquent cortex and/or subcortical language pathways, who underwent surgical resection. Eligibility was defined by tumour entity rather than by grade. Adult-type diffuse glioma, as defined by the WHO Classification of Tumours of the Central Nervous System, comprises astrocytoma IDH-mutant (grades 2–4), oligodendroglioma IDH-mutant and 1p/19q-codeleted (grades 2–3) and glioblastoma IDH-wildtype (grade 4). All tumour grades within this spectrum were eligible, and no study was excluded solely on the basis of tumour grade. However, the database search was more restrictive than this general definition: the population criteria emphasised grades 2 and 3, and a NOT operator filtered out records whose titles or abstracts included terms like “glioblastoma,” “GBM,” “gliosarcoma,” “grade 4,” “high grade,” or “HGG.” This approach aimed to prioritise lower-grade diffuse gliomas, where awake language mapping is most commonly reported. Consequently, this may have limited the retrieval of eligible higher-grade or mixed-grade cohorts, which is acknowledged as a limitation related to search sensitivity rather than eligibility. When language outcome data could not be separated by grade, cohorts spanning multiple grades were retained. Therefore, the combined population covers WHO grades 2 to 4. Since the included studies range across the 2007, 2016, and 2021 WHO classifications, tumour grade is consistently reported using Arabic numerals (grades 2–4), with any differences in grading systems noted as reported by the original authors. Studies were included only if they involved awake craniotomy with direct electrical stimulation (DES) for language mapping during resection, aimed at identifying language-eloquent areas.

Both randomised and non-randomised studies were eligible for inclusion. These encompassed randomised controlled trials, case series, prospective, and retrospective cohort studies. Conference abstracts, case reports, letters, and studies not published in English were excluded.

### Study selection

All records retrieved from database searches were compiled into Rayyan, where duplicates were removed [10]. Three reviewers, MN, PM, and EM, independently screened titles and abstracts to identify potentially relevant studies, and then conducted full-text reviews to confirm eligibility against predefined criteria. Any disagreements among the reviewers were resolved through consensus.

### Data extraction

Data from each included study were independently extracted by two reviewers, AY and MB, using a standardised data extraction template. Any discrepancies were resolved through discussion and consensus. The extracted data included study characteristics such as author, publication year, country, study design, and sample size. Details about the population were also recorded. The main outcomes of interest were postoperative language deficits following awake craniotomy with direct electrical stimulation (DES) language mapping, early postoperative language outcomes (within 3 months), and late postoperative language outcomes (3 months or more), where reported.

### Risk of bias assessment

The methodological quality of the included studies was assessed using the Joanna Briggs Institute (JBI) Critical Appraisal Checklists for case series and cohort studies [11]. Two reviewers (MN and PM) independently assessed each study, resolving any disagreements through discussion until they reached consensus. The JBI checklists do not provide a prescribed algorithm for combining individual domain responses into an overall summary judgement. Instead, reviewers reached their final ratings through a consensus process, considering the likely impact of each domain on the validity of the language outcomes rather than treating all flagged domains equally. Greater importance was given to factors like complete and consecutive participant inclusion, valid and reliable outcome measurement, thorough follow-up, and appropriate statistical analysis, since these aspects most directly influence whether the reported deficit rates truly reflect the source cohort. If a critical domain was judged inadequate, the study was rated as high risk even if only a few other domains were flagged overall. Conversely, studies with several unclear or inadequate domains, especially those less relevant to outcome determination, were rated as moderate risk. These overall ratings served mainly for descriptive purposes and were not used to weight or exclude studies in the synthesis. Afterwards, the risk-of-bias assessments were visualised using the RobVis application [12].

### Data synthesis

Due to considerable clinical and methodological heterogeneity among the included studies covering variations in study design, patient selection, tumour characteristics, surgical techniques, definitions of language deficits, assessment methods, and follow-up durations, a meta-analysis was deemed inappropriate. Instead, a narrative synthesis was performed. The studies were organised into tables and summarised by key features, then grouped by the timing of postoperative language evaluation. Language outcomes were analysed across three prospectively defined categories. Early deficits were assessed at any timepoint from immediately postoperatively up to and including 7 days. Late deficits were assessed at 3 months or later. Persistent deficits were defined as language impairment documented at the latest available assessment at 3 months or later. Assessments between 8 days and less than 3 months (including the 2-week and 1-month timepoints reported in two studies) fell outside these definitions and are reported separately as intermediate assessments rather than pooled with late or persistent outcomes. Where a study reported multiple qualifying timepoints, the specific timepoint is stated alongside each figure. When available, the nature of the language impairments and patterns of recovery were also summarised. The findings were interpreted descriptively, with particular attention to differences in assessment approaches and reporting practices across studies.

Assessment of reporting bias was initially planned but could not be conducted quantitatively. Funnel plot inspection and statistical tests for small-study effects require a pooled estimate from at least around ten comparable studies, which was not possible due to heterogeneity in outcome definitions and assessment timepoints. Consequently, reporting bias was discussed narratively, considering the dominance of small single-centre case series, the lack of prospective protocol registration in most included studies, and the possibility that studies with unfavourable language outcomes are less likely to be published. Similarly, the certainty of evidence was assessed narratively rather than through formal GRADE rating, since domains like inconsistency, imprecision, and indirectness cannot be operationalised without a pooled effect estimate. Based on this, the overall certainty of evidence for both early and persistent language deficits was judged to be very low because of the observational nature of the studies, small sample sizes, heterogeneous outcome assessments, and potential reporting bias. The conclusions are therefore framed accordingly.

## Results

**Fig. 1:**
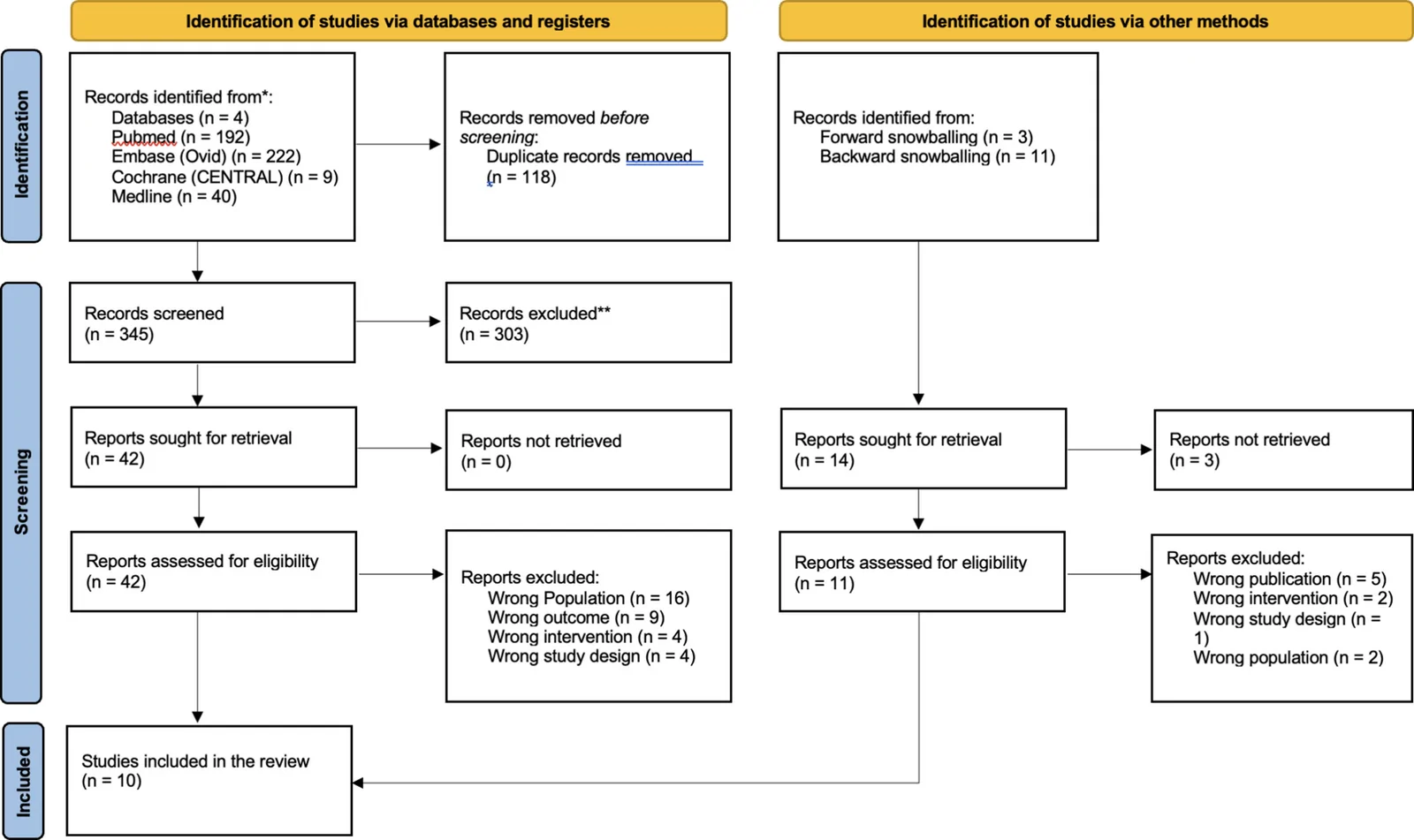
PRISMA flow diagram illustrating the study selection process from databases exportation to included studies after full text screening

The literature search across the four databases (PubMed, Embase via Ovid, MEDLINE, and Cochrane CENTRAL) yielded 463 records. Following importation to Rayyan, 118 duplicates were detected and subsequently removed. Three hundred forty-five records underwent title and abstract screening; 42 records were included for full text retrieval and underwent full text screening, resulting in the 10 included records for analysis (Fig. 1, Table 1).

**Table 1:** Table of study characteristics. Summary of study design, baseline patient demographics and tumour characteristics, followed by direct electrical stimulation (DES) mapping approach and language assessment method used within preoperative and postoperative follow-up of awake craniotomies on diffuse gliomas in language-eloquent areas

Study	Study design	Study population (total n. patients; *n*(%)male, *n*(%)female)	Tumour grade, *n*	Tumour location,* n* (%)	DES mapping type	Language assessment method	Follow up duration
Chang et al. [13]	Prospective observational study	19; 9(47.4%) male, 10(52.6%) female	II 7(36.8%), III 8(42.1%), IV 2(10.5%)	Frontal 12(64%), parietal 1(5%), frontotemporal 3(16%), frontoparietal 1(5%), fronto-insula 1(5%), Insula 1(5%)	Cortical	sfBDAE, DO80, PPTT	3 months
Kahana et al. [14]	Prospective observational study	28; 16(57%) male, 12(43%) female	II 12(42.9%), III 1(3.57%), IV 15(53.6%)	Frontal 3, parietal 8, temporal 5, frontoparietal 1, insula 5, SMA 6	Cortical and subcortical	Comprehensive language battery (picture naming, Reading, Sentence repetition, Syntax tasks	3–6 months
Li et al. [15]	Retrospective cohort study	Total: 225; 116(51.6%) male, 109(48.4%) female. MAC (subgroup): 184; 95(51.6%) male, 89(48.4%) female. AAA (subgroup): 41; 21(51.2%) male, 20(48.8%) female	MAC: I 14(7.6%), II 58(31.5%), III 37(20.1%), IV 75(40.8%). AAA:I 0(0.0%), II 17(41.5%), III 11(26.8%), IV 13(31.7%)	AAA: frontal 14 (34.1%), temporal 3 (7.3%), parietal 8 (19.5%), two or more brain lobes 16 (39.0%); MAC: frontal 54 (29.3%), temporal 25 (13.6%), parietal 18 (9.8%), two or more brain lobes 46 (25%)	Cortical	WAB, MRS	6 months
Papagno et al. [16]	Prospective observational cohort study	44; 26(59.1%) male, 18(40.9%) female	Low-grade (I, II) 25(56.8%), high-grade (III, IV) 19(43.2%)	Frontal and temporal (frontal UF removal *n* = 8; temporal UF removal *n* = 10; no UF removal *n* = 26)	Cortical and subcortical	Language tasks: verbal fluency; naming of famous faces; picture naming of objects and actions; naming by description; pointing to picture; sentence comprehension and picture-to-sentence matching task; repetition of syllables, words, non-words and sentences	3 months
Saito et al. (2016) [17]	Retrospective observational study	Total: 82; 48(58.5%)male, 34(41.5%) female. PT(subgroup): 18; 9(50%)male, 9(50%) female. No PT(subgroup): 64; 39(60.9%)male, 25(39.1%) female	II 45(54.9%), III 27(32.9%), IV 10(12.2%)	Frontal 82(100%) (dominant hemisphere, PT subgroup analysed)	Cortical and subcortical	Naming, reading, verb generation, spontaneous speech monitoring (through evaluation of recall, counting, fluency, and comprehension)	6 months
Saito et al. (2022) [18]	Prospective case series	7; 5(71.4%) male, 2(28.6%) female	III 5(71.4%), IV 2(28.6%)	Frontal 7(100%)(frontal operculum, middle frontal gyrus, inferior frontal gyrus, precentral gyrus)	Cortical and subcortical	Language function assessed by speech therapist (naming, repetition, spontaneous speech)	24 months
Sellier et al. [19]	Retrospective cohort study	Total 84; 48(57.1%) male, 36(42.9%) female. Group 1: 63; 33(52.4%) male, 30(47.6%) female. Group 2: 21; 15(71.4%) male, 6(28.6%) female	Low-grade 84(100%)	NR	Cortical and subcortical	evaluation of spontaneous speech, a semantic fluency task, spoken language tested by DO80 (picture naming task) in native language, speech articulation tested by counting 1–10 in native language	Mean 42.3 ± 33.5 months
Suarez-Meade et al. [20]	Retrospective consecutive case series	15; 10(66.6%) male, 5(33.3%) female	Low-grade(I, II) 3(20%), high-grade(III, IV) 12(80%)	Frontal 15 (100%), temporal 1 (6.7%), insular 8 (53.3%)	Cortical and subcortical	Tablet-based NeuroMapper (picture naming paradigm)	3 months
Tsuzuki et al. [21]	Retrospective case series	18; 12(66.67%) male, 6(33.33%) female	NR	Frontal and temporal lobe	Cortical and subcortical	IEMAS-naming, reading, repetition, SLTA, clinical assessment (post operatively)	Mean 114.8 months
Vassal et al. [22]	Prospective case series	10; 4(40%) male, 6(60%) female	I 1(10%), II 3(30%), IV 6(60%)	Frontal 3 (30%), temporal 4 (40%), parietal 2 (20%), fronto-insular 1 (10%)	Cortical and subcortical	Picture naming (DO80, Intraop), NIHSS post operatively	1 month

*Abbreviations*: *AAA* Asleep-Awake-Asleep, *DES* direct electrical stimulation, *DO80* Deloche 80 Naming Test, *IEMAS* Intraoperative Examination Monitoring for Awake Surgery, *MAC* Monitored Anaesthesia Care, *MRS* Modified Rankin Scale, *NIHSS* National Institutes of Health Stroke Scale, *NR* not reported, *PPTT* Pyramids and Palm Trees Test, *PT* pars triangularis, *sfBDAE* Short-form Boston Diagnostic Aphasia Examination, *SLTA* Standard Language Test of Aphasia, *SMA* Supplementary Motor Area, *UF* uncinate fasciculus, *WAB* Western Aphasia Battery

### Risk of bias

**Fig. 2:**
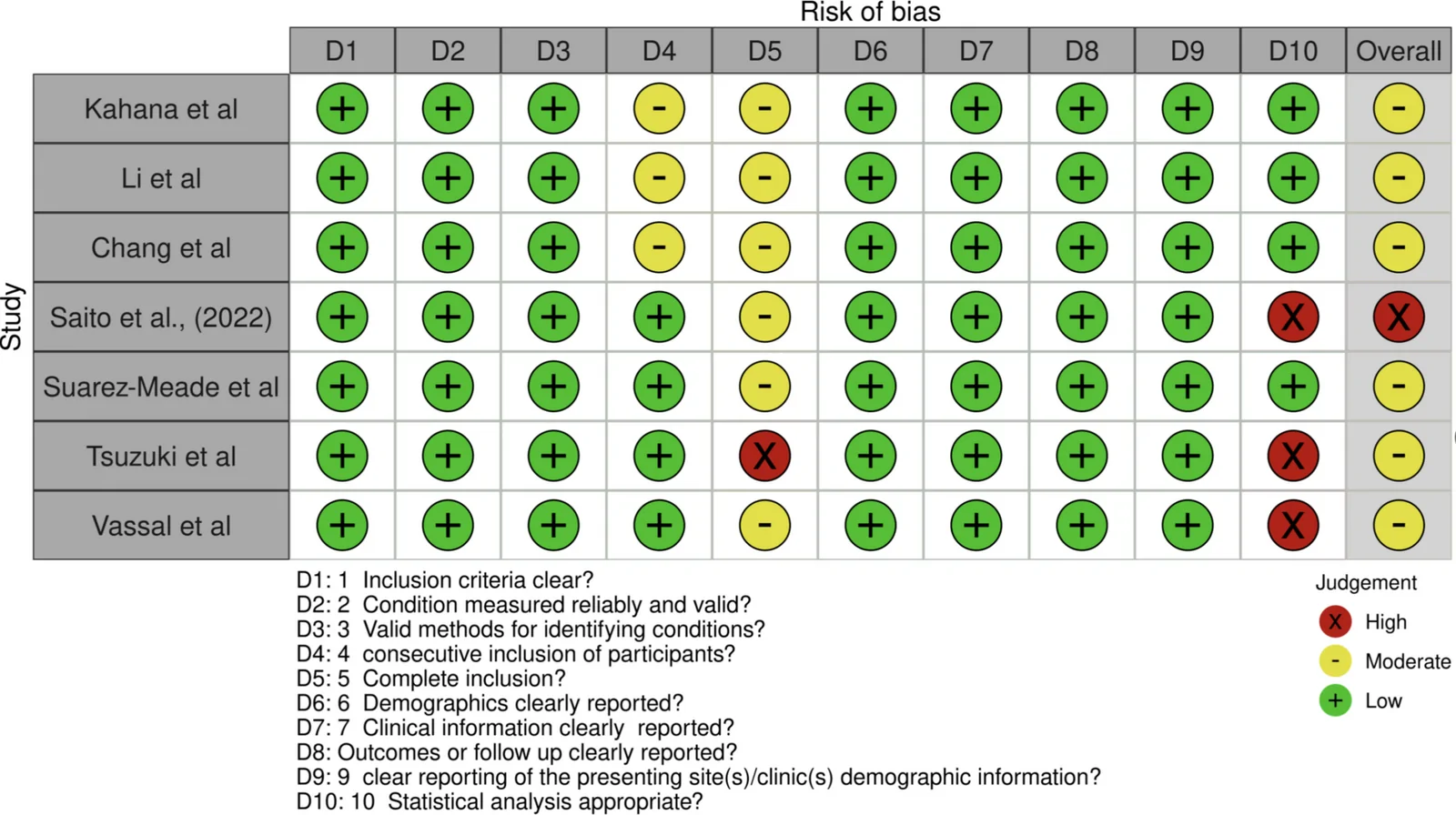
Risk of bias for the seven case series assessed with the JBI Critical Appraisal Checklist for case series. Traffic light plots generated with RobVis present domain-specific and overall risk of bias assessments for each study. Most domains were rated “yes” (green), indicating low risk of bias across several key methodological areas. Inadequate or unclear ratings clustered in D4 (consecutive inclusion of participants), D5 (complete inclusion) and D10 (appropriateness of statistical analysis). Six of the seven case series were judged to be at moderate overall risk of bias and one (Saito et al. 2022) at high risk

**Fig. 3:**
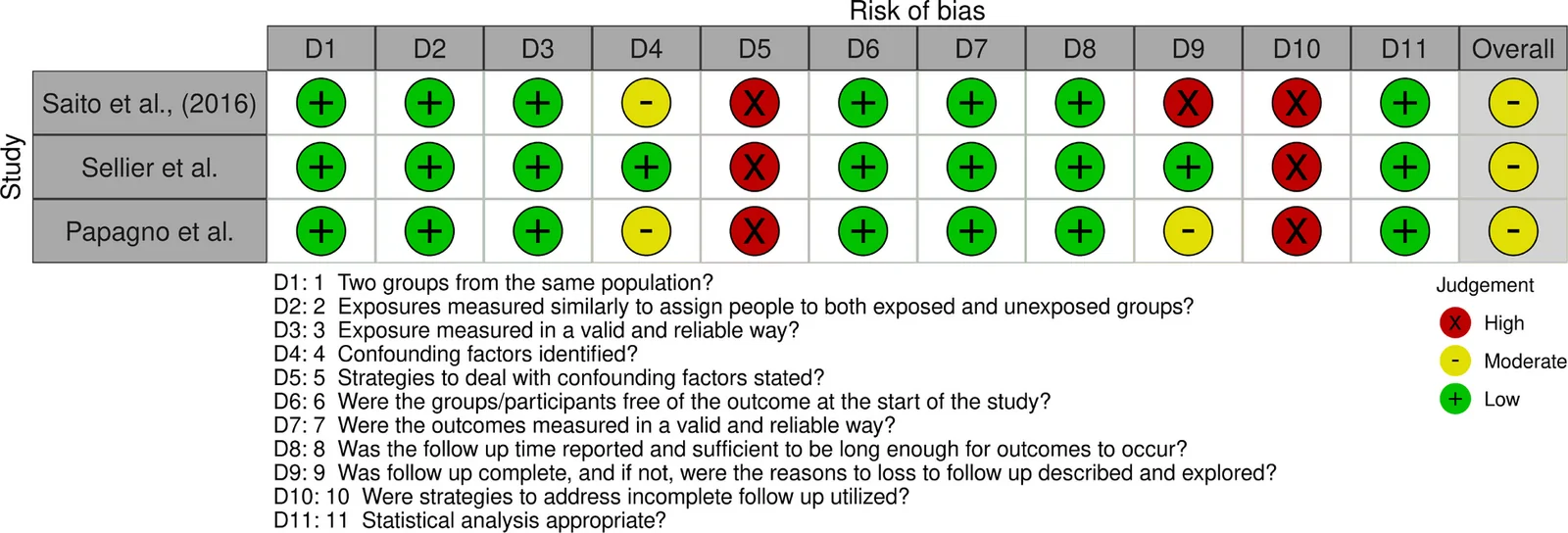
Risk of bias for the three cohort studies assessed with the JBI Critical Appraisal Checklist for cohort studies. Traffic light plots generated with RobVis present domain-specific and overall risk of bias assessments for each study. Most domains were rated “yes” (green), indicating low risk of bias across several key methodological areas. Inadequate or unclear ratings clustered in D5 (strategies to deal with confounding factors), D9 (completeness of follow-up and reporting of losses) and D10 (strategies to address incomplete follow-up), with D4 (identification of confounding factors) unclear in two studies. All three cohort studies were judged to be at moderate overall risk of bias

The risk of bias was assessed to evaluate the validity of the included studies. This is shown in Figs. 2 and 3, where traffic light plots generated by RobVis summarise judgments for each domain across the included studies. Figure 2 presents the seven studies appraised with the JBI case series checklist (10 domains) and Fig. 3 the three studies appraised with the JBI cohort checklist (11 domains), corresponding to the study design classification above. For the case series, most domains were rated “yes” (green), indicating a low risk of bias in key areas, including clearly defined inclusion criteria, valid measurement of conditions, and comprehensive reporting of demographics and clinical details. Overall, the case series demonstrated low, moderate, or high risk of bias depending on the study.

### Clinical outcomes of the included studies

#### Study characteristics

In this review, the results of 10 studies are included. The studies were published between 2011 and 2025. Of these studies, 3 were appraised as cohort studies using the JBI cohort checklist (Saito et al. 2016, Papagno et al. and Sellier et al.) [16, 17, 19], and 7 were appraised as case series using the JBI case series checklist (Chang et al., Kahana et al., Li et al., Saito et al. 2022, Suarez-Meade et al., Tsuzuki et al., and Vassal et al.) [13–15, 18, 20–22]. A total of 532 patients undergoing awake craniotomy were included in the 10 studies, with the sample sizes ranging from 7 to 225. Most of the tumours were in the frontal lobe, while some of the studies reported temporal, parietal and insular involvement. Overall, 294 (55.3%) patients were male. WHO grade 4 tumours were represented in at least six of the ten cohorts, most substantially in Kahana et al. (15/28 glioblastomas), Vassal et al. (6/10), and Saito et al. (2022) (2/7), with further glioblastomas in Tsuzuki et al., Saito et al. (2016), and Chang et al.

The included population spans the adult-type diffuse glioma spectrum, WHO grades 2 to 4. Tumour grade was reported with differing levels of detail across studies, and a pooled grade distribution could not be derived reliably. Where full grade breakdowns were available, higher-grade tumours predominated: Li et al. reported 88/225 WHO grade 4 and 48/225 grade 3 (136/225, 60.4% higher-grade), Vassal et al. 6/10 grade 4, Kahana et al. 15/28 glioblastomas, and Saito et al. (2022) 7/7 higher-grade including 2 grade 4. Papagno et al. reported 25/44 lower-grade and 19/44 higher-grade, and Chang et al. 53% grade 3–4. Sellier et al. described all patients as having diffuse lower-grade glioma without stating WHO grade. At least 114 patients across the included cohorts had WHO grade 4 tumours. Grades are reported using current WHO CNS nomenclature; where original authors used historical terminology (grade I–IV, or “low-grade glioma” without a stated grade), the reported category has been mapped to the nearest current equivalent. It is important to note that Sellier et al. reported that all included patients had low-grade gliomas but did not explicitly report WHO grade, Tsuzuki et al. reported histopathology for all 18 patients, including two glioblastomas, spanning WHO grades 2–4 [19, 21].

### Language assessment tools

Language assessment varied noticeably across the 10 studies included. Several studies used standardised tests such as the short-form Boston Diagnostic Aphasia Examination (sfBDAE), Deloche Orale 80 (DO80) and Western Aphasia Battery (WAB), while others assessed the language outcomes using more informal assessments such as picture reading, naming and word generation. Of note, the only study to use the NIH stroke scale to measure postoperative language outcomes was Vassal et al. [22]. Of the studies which used a formal language assessment, only 2 used the same tool [13, 19]. Tsuzuki et al. assessed language postoperatively using the Standard Language Test of Aphasia (SLTA), administered by a speech therapist, with evaluation of syntactic distortion, dysarthria, and aphasia [21]. Direct comparability of the studies is limited due to the heterogeneity of the assessment methodologies. The baseline language status, the type and timing of intraoperative stimulation-induced disturbances, the cortical or subcortical location of the functional boundary, the influence of these findings on the decision to continue or halt resection, and the extent of resection achieved are summarised for each study in Table 2. Preoperative language impairment was common where formally assessed, affecting 6 of 10 patients in Vassal et al. and 14 of 15 in Suarez-Meade et al., and the extent of resection was quantified in 8 of the 10 studies.

**Table 2:** Baseline language status, intraoperative mapping findings, functional boundary, influence on resection, and extent of resection across the included studies

Study	Baseline language status	Intraoperative disturbance: type and timing	Functional boundary (cortical/subcortical)	Influence on decision to continue or stop resection	Extent of resection
Chang et al. [13]	DO 80 and PPTT performed preoperatively in all patients. sfBDAE assessed preoperatively and at 1 week	Naming errors on DO 80 or incorrect PPTT responses during cortical mapping and continuous monitoring throughout resection	Cortical language-positive sites in 10/19 (52%), mainly left middle and inferior frontal gyri	Excision stopped when naming errors appeared on DO 80 or responses were incorrect on PPTT	Total resection (> 95%) in 11/19 (58%) and subtotal (85–95%) in 3/19 (16%)
Kahana et al. [14]	Preoperative language assessment in all patients. Deficits documented per patient, including dyslexia, syntax, naming and phonological buffer deficits, with intraoperative tasks adjusted to baseline ability	Subcortical DES-evoked language interference, with threshold defined as the minimal intensity producing interference (2–20 mA). No interference evoked in 11/32 (34.4%) cases	Subcortical AF, IFOF, ILF, SLF, and FAT targeted. Distance from stimulation point to nearest tract measured against preoperative DTI	Threshold-based proximity alerts used to guide resection towards language tracts. No fixed stopping rule stated	NR
Li et al. [15]	KPS and MMSE recorded preoperatively, with MMSE 28–30 in 93.5% of MAC and 95.1% of AAA patients. Pre- and postoperative neurological function assessed	Cortical mapping with object naming, verb generation, counting and reading. Speech arrest elicited at positive sites	Cortical electrode mapping of language sites (e.g. left inferior frontal gyrus, Broca area)	Resection continued until brain function was affected, as judged by cortical electrode monitoring or communication with the patient	MAC total 117/184 (63.6%), subtotal 52 (28.3%), partial 15 (8.1%). AAA total 19/41 (46.3%), subtotal 15 (36.6%), partial 7 (17.1%) (*p* = 0.076)
Papagno et al. [16]	Full neuropsychological battery performed preoperatively. Groups did not differ before surgery	Cortical and subcortical DES. Temporal UF stimulation induced phonemic paraphasias, and frontal UF stimulation induced phonemic paraphasias followed by speech arrest	Subcortical uncinate fasciculus (frontal and temporal portions) and IFOF, confirmed by pre- and postoperative DTI-FT	Positive sites required a reproducible error on three or more stimulations and were tagged. UF removal determined by tumour involvement	NR
Saito et al. (2016) [17]	Preoperative speech assessment performed. Only 1/18 had mild dysphasia	Cortical stimulation interrupting, disturbing or slowing picture naming, word reading or verb generation, with each site tested three or more times	Cortical FLA at pars opercularis and pars triangularis. FLA not identifiable in 11/18 (61%) with PT involvement. Subcortical stimulation used during resection	Resection stopped in the illustrative case when transcortical MEPs fell below 60% of pre-removal amplitude	Mean 90% ± 7.1% (PT-involved subgroup)
Saito et al. (2022) [18]	Two of seven had mild preoperative speech disturbance (dysarthria or dysphasia). The remaining five were normal	Intraoperative naming failure, repetition failure, mild dysarthria and mild paraphasia. CCEP monitored continuously during removal	Cortical FLA identified by mapping. CCEP recorded FLA to PLA to monitor language-related fibres, with subcortical mapping also used	CCEP change during deep tumour removal accompanied observed language dysfunction and was used to predict postoperative recovery	Mean 93% (range 85–98%)
Sellier et al. [19]	Standard neuropsychological assessment performed preoperatively by the same assessors, comprising DO 80, semantic fluency and spontaneous speech	Transitory disturbances induced in the native language of all patients. Interpreter identified semantic and phonological paraphasia, dysarthria or anarthria, perseveration and anomia	Cortical and subcortical electrostimulation mapping	Resection tailored to cortico-subcortical functional boundaries. Language barrier noted as potentially limiting extent of resection	Mean 90% ± 10.0. Total resection 25%, subtotal 59% and partial 16% in group 1
Suarez-Meade et al. [20]	Fourteen of 15 (93.3%) presented with a preoperative speech deficit, comprising non-fluent aphasia (*n* = 5), verbal working memory deficits (*n* = 8) and speech arrest (*n* = 4)	Errors in picture naming during cortical stimulation in 10/15 (66.7%). Two of 15 (13.3%) declined in naming during resection, exceeding a 5% error rate versus baseline	Cortical vPMC (60%), pars triangularis (20%) and pars opercularis (20%). Subcortical SLF, IFOF and AF positive for speech arrest and phonetic paraphasia	Resection stopped in patients presenting with any deficit to preserve language function. Residual tumour attributed to language preservation in five cases	Mean 60.35% ± 29.60. GTR 4/15 (26.6%), STR 5/15 (33.3%) and PR 6/15 (40%)
Tsuzuki et al. [21]	Preoperative fMRI used to determine language dominance. SLTA administered postoperatively by a speech therapist	Speech arrest on cortical stimulation, with NMA distinguished from FLA by impaired fast alternating tongue movements. Apraxia of speech appeared during resection in all four NMA-resected cases	Cortical NMA localised inferior to the precentral gyrus in all cases and distinguished from FLA. Subcortical stimulation performed in deep white matter	Cortical areas not infiltrated by tumour were spared. NMA resected only where tumour invaded the same gyrus	Mean 94.4% in three of four NMA-resected cases. Partial resection in one case due to prolonged seizures
Vassal et al. [22]	Six of 10 (60%) had preoperative language deficits on NIHSS item 9, comprising mild motor or sensorimotor aphasia. Outcomes reported relative to baseline per patient, with deficits in 60% preoperatively, 80% at 24 h and 50% at 1 month, comprising new deficits in 20% and worsening of pre-existing deficits in 10%, all reversible within 30 days	Transient language disturbance during DO 80 naming with cortical and subcortical DES. Disturbance type detailed by a speech therapist	Cortical eloquent areas tagged to define superficial resection boundaries. Subcortical AF, IFOF, ILF, UF, SubCF, and premotor fibres reconstructed by DTI-FT	Subcortical sites deemed essential when three or more DES induced language inhibition with normalisation on cessation, defining the deep resection limit	EOR 93.0% ± 10.4 by relative volumetric analysis, with gross total resection in 60%. Mean tumour volume reduced from 43.4 ± 23 cm^3^ to 4.3 ± 7.4 cm^3^

*Abbreviations*: *AF* arcuate fasciculus, *CCEP* cortico-cortical evoked potentials, *DES* direct electrical stimulation, *DO 80* Deloche 80 naming test, *DTI-FT* diffusion tensor imaging fibre tracking, *EOR* extent of resection, *FAT* frontal aslant tract, *FLA* frontal language area, *fMRI* functional magnetic resonance imaging, *GTR* gross total resection, *IFOF* inferior fronto-occipital fasciculus, *ILF* inferior longitudinal fasciculus, *KPS* Karnofsky Performance Status, *MEP* motor evoked potential, *MMSE* Mini-Mental State Examination, *NIHSS* National Institutes of Health Stroke Scale, *NMA* negative motor area, *PLA* posterior language area, *PPTT* Pyramids and Palm Trees Test, *PR* partial resection, *PT* pars triangularis, *sfBDAE* short-form Boston Diagnostic Aphasia Examination, *SLF* superior longitudinal fasciculus, *SLTA* Standard Language Test of Aphasia, *STR* subtotal resection, *UF* uncinate fasciculus, *vPMC* ventral premotor cortex

### Early postoperative outcomes

Except for Kahana et al., every study carried out early postoperative language assessment [14]. Earliest assessment timing was conducted immediately post-op and latest early assessment timing was 1 week post-operation. Notably, two studies did not report the number of patients with early language deficit [13, 14].

The number of patients with early postoperative deficits varied across studies, with some having higher (80%) and some having lower deficits (13.33%) [20, 22]. In one study, all the patients showed early postoperative language deficit [18]; while other studies reported that a minority of their patients experienced early postoperative deficits [17, 20]. Importantly, the timing of assessment may influence reported deficit. In one study, a smaller proportion of patients who were assessed later (1 week post-op) were reported to have deficits (4/18, 22%), whereas, in other studies all of the patients who were assessed immediately postoperatively showed deficits (7/7, 100%) [17, 18]. However, comparisons as such should be made cautiously given the considerable difference in sample sizes (7 vs 18 patients) [17, 18].

The reported deficits spanned heterogeneous linguistic phenomena, and most studies grouped them, so the persistence of individual deficit types could not be analysed. Within this constraint, the studies reported a broadly similar spectrum of early postoperative language deficits. The most common deficits reported were naming errors, motor aphasia and repetition errors [14, 17, 22]. Of note, Tsuzuki et al. reported ‘Apraxia of Speech’ as the early language deficit from their study [21].

### Late outcomes and persistent deficits

Postoperative language outcomes beyond the early window were reported in 6 studies, with assessment timepoints ranging from 2 weeks to 24 months. Applying the prespecified definitions, four studies provided assessments at ≥ 3 months and therefore contributed to the persistent deficit synthesis [15–17, 19]. Two studies reported only intermediate assessments, Vassal et al. at 1 month [22] and Tsuzuki et al. at 1–2 months [21], which fall short of the 3-month threshold and are reported separately as intermediate outcomes. Saito et al. (2022) followed patients individually to recovery over 0.5–24 months, with all seven recovering [18], and Suarez et al. assessed patients at 2 weeks and at 3 months [20]. Most studies reported a reduction in language deficits at late follow-up compared to early postoperative assessment, suggesting a pattern of recovery over time.

Among the four studies assessing patients at ≥ 3 months, Li et al. reported no persistent language deficits at 3 months [15]. Two further studies reported complete resolution of deficits, but only at intermediate timepoints that fall short of the 3-month threshold: Vassal et al. at 1 month, and Tsuzuki et al. at 1–2 months, the latter applying only to the 4 patients who underwent NMA resection [21, 22]. Across the four studies providing assessment at ≥ 3 months, reported rates ranged from 0 to 47% depending on the outcome measure and subgroup [15–17, 19]. These fall into two distinct groups. In three studies reporting whole-cohort outcomes with unselected tumour locations, persistent language deficits were 0–5.6%, the upper bound arising from a single patient in Saito et al. (2016) (1/18); Li et al. and Sellier et al. reported no persistent language deficits (0/225 assessed at 90 days and 0/81 at 1 year respectively) [15, 17, 19]. In the fourth, Papagno et al. assessed 44 patients at a single 3-month follow-up using task-level neuropsychological cut-offs rather than a global aphasia measure, and reported outcomes stratified by uncinate fasciculus status: 8/17 (47%) of patients with uncinate resection scored below cut-off on famous face naming, compared with 2/20 (10%) of those with the tract preserved (pooled, 10/37, 27%) [16]. This figure is reported separately rather than excluded from the overall range, but is not directly comparable with the whole-cohort rates above: it reflects impairment on a single proper-naming task in a cohort selected for anterior frontal and temporal tumours, but not a general persistent language deficit rate following awake craniotomy. Suarez et al. reported that recovery relative to baseline was observed in all 15 patients by 2 weeks, although 7/15 (46.7%) still had mild residual speech and language deficits at that point, comprising mild expressive aphasia, word-finding difficulty, slower auditory naming and dysarthria [20]. This figure reflects incomplete recovery at 2 weeks rather than a persistent deficit. Interpretation is further complicated by the fact that 14/15 patients (93.3%) presented with a preoperative speech deficit, so postoperative findings cannot be interpreted as de novo impairment. The separate 13.3% figure refers to two patients with a mild decline in naming detected during intraoperative monitoring, not to a postoperative assessment [20]. Sellier et al. (*n* = 84) reported permanent neurological deficits in 3/81 (3.7%) at 1 year; however, none of these were language deficits, comprising a lower-limb proprioceptive deficit and dysesthesias in two patients, such that the persistent language deficit rate in this cohort was 0/81 (0%) [19]

### Recovery patterns

The overall pattern of recovery across studies was early deterioration followed by substantial improvement over time. Most early postoperative deficits resolved within 3 months, with several studies reporting near complete recovery by 6 months [15, 17]. Of note, Vassal et al. reported complete resolution of all deficits within 1 month [22]. The rate and extent of recovery varied between studies, with one patient in Saito et al. (2022) requiring 24 months to recover, this being the patient with the greatest intraoperative decline in cortico-cortical evoked potentials; all seven patients in that cohort ultimately recovered [18]. Prolonged recovery was also reported by Tsuzuki et al., in whom apraxia of speech following negative motor area resection persisted postoperatively and was judged near-recovered at a mean of 8.7 months; by contrast, working memory and verbal fluency recovered within 3 months, and all four patients returned to work at a mean of 5.5 months [21]. It is important to note that although Suarez et al. reported that 7/15 (46.7%) patients showed long-term deficit at late assessment compared to 2/15 (13.3%) patients who showed deficit at early assessment (intraoperative assessment), they reported that ‘all patients improved compared to baseline by 2 weeks’ [20].

## Discussion

This systematic review demonstrates that early postoperative language deficits following awake craniotomy for diffuse gliomas in language-eloquent areas are common but highly variable, ranging from 2.2 to 100% across the included studies depending on assessment timing and measure. However, when assessed at ≥3 months using whole-cohort language measures, persistent language deficits were low, ranging from 0 to 5.6%, with the upper bound arising from a single patient in Saito et al. (2016) [15, 17, 19]. Studies such as Papagno et al. and Suarez et al. reported higher rates in specific subgroups, with 8/17 (47%) of patients undergoing uncinate fasciculus resection impaired on famous face naming at 3 months and residual mild deficits in 46.7% at 2 weeks respectively, making them apparent outliers; in the latter, all patients had nonetheless improved relative to baseline by that point, and 14/15 had a preoperative speech deficit [16, 20]. An important signal to consider is not the variability in early deficits but a consistent recovery trajectory over time.

One important finding from this review is that the reported incidence of postoperative language impairment tends to vary systematically depending on the timing of assessment. Because the included studies differed considerably in design, population, tumour grade, assessment tools, and follow-up schedules, this pattern cannot be distinguished from confounding factors. Therefore, no definitive causal conclusions can be drawn from a primarily observational narrative synthesis. This is observed in studies assessing patients immediately postoperatively reporting substantially higher deficit rates, such as 80% in Vassal et al. and 100% in Saito et al. (2022), relative to studies assessing patients at later timepoints (e.g. 22% in Saito et al. (2016) and 14.6% in Li et al.) [15, 17, 18, 22]. These results may indicate that early postoperative deficits reflect transient functional disruption, possibly due to temporary network dysfunction and oedema. Therefore, “early deficit rates” should not be correlated with fixed outcome measures, and comparing studies without accounting for assessment timing may be potentially misleading. This calls for standardised early assessment timepoints in future studies, as it has important implications for patients.

The available data also point to subcortical white matter pathways as a potentially important contributor to postoperative language outcomes, although the evidence base is limited to a small number of observational cohorts. In 44 patients undergoing awake surgery for left frontal or temporal glioma, Papagno et al. found impairment mainly in those with uncinate fasciculus resection: at 3 months, 8/17 (47%) scored below cut-off on face naming versus 2/20 (10%) with tract preservation, with significance only at follow-up. This suggests that the elevated deficit rate represents a tract-specific subgroup signal rather than the expected persistent deficit rate after awake craniotomy more broadly. This supports the growing emphasis on subcortical mapping, specifically within the framework of the connectomic approach to glioma surgery, where preservation of the critical white matter tracts is a priority (Duffau paradigm) [23]. Hence, the variability of the postoperative outcomes across studies may reflect differences in tumour proximity to key language pathways such as the inferior fronto-occipital fasciculus and arcuate fasciculus, instead of differences in surgical technique alone. This observation suggests that future studies should stratify outcomes based on white matter tracts involvement to have a better understanding of the functional risks.

### Strengths and limitations

This systematic review has several strengths, including pre-registration to PROSPERO, independent triple screening, and formal risk of bias assessments using the JBI tools for case series and cohort studies. In addition, this review differs uniquely from the previously published literature. The scope and focus in Collée et al., for instance, were examining intraoperative language errors and predictive values for postoperative outcomes, but not the direct synthesis of postoperative language deficits following awake craniotomy [24]. Similarly, a major study by Sanai et al. provided important single-centre evidence supporting awake mapping but did not involve a systematic synthesis of the outcomes [8]. Contrastingly, this systematic review evaluates postoperative language outcomes following awake craniotomy in diffuse glioma patients, with explicit differentiation between early and late deficits. This addresses an important gap in the literature by allowing for a clearer understanding of the recovery dynamics.

However, limitations must be acknowledged. The synthesis was narrative due to significant heterogeneity in timings of both early and late assessments and inconsistency in outcomes reported. A small number of studies (*n* = 10) contributed to the results, and formal quantitative assessment of reporting bias was not feasible given the heterogeneity of outcomes and the number of comparable studies. Reporting bias cannot be ruled out and was judged, on narrative grounds, to be a plausible concern, particularly given the predominance of small, single-centre case series and the likely underreporting of unfavourable outcomes. Taken together with the observational study designs and heterogeneous outcome ascertainment, the overall certainty of the evidence was judged to be very low, and the findings should be interpreted as descriptive signals rather than reliable effect estimates. The restriction to English-language studies may have excluded relevant data that could have highlighted other key postoperative outcomes. The search architecture is a further limitation: records were required to satisfy a comparator or adjunctive-technique block as a mandatory AND condition, which will have reduced sensitivity for single-arm studies reporting awake DES language mapping alone. Forward and backward citation searching of included studies, relevant reviews and citing articles was undertaken to mitigate this and identified additional eligible records. The same constraint applies to tumour grade: although eligibility was defined by tumour entity and grade 4 diffuse gliomas were eligible throughout, the search string’s exclusion of glioblastoma and high-grade descriptors likely reduced the retrieval of eligible higher-grade cohorts. Grade 4 tumours still comprise at least 114 of the 532 included patients. Therefore, the findings should be interpreted as applicable across the entire diffuse glioma spectrum, but not limited to lower-grade tumours. Another limitation is that postoperative outcomes were not consistently compared to preoperative language status. Few studies included formal preoperative assessments, making it difficult to judge postoperative changes. For example, Vassal et al. noted mild aphasia in 6 out of 10 patients before surgery, so the reported 80% early deficit rate cannot be considered a new impairment. As a result, it is challenging to differentiate between new deficits, worsening of existing impairments, and persistent or improved baseline issues across the studies. Additionally, the reported deficits are diverse, including aphasia, anomia, paraphasia, dysarthria, apraxia of speech, and repetition failure, which were usually grouped together rather than reported separately. This makes it hard to determine which specific deficits are most likely to persist. Nonetheless, one other major limitation is the substantial heterogeneity in language assessment methodologies across studies. This is highlighted in studies such as Li et al. and Chang et al. using validated tools such as Western Aphasia Battery and Boston Diagnostic Aphasia Examination, respectively, whilst others relied on non-standardised tools, including naming tasks and fluency measures (Table 3). The overall heterogeneity, therefore, resulted in the preclusion of the quantitative synthesis and emphasised the need for standardisation in outcome reporting.

**Table 3:** Summary of the results table: Early and late postoperative language deficits and recovery pattern following awake craniotomy with direct electrical stimulation mapping for diffuse gliomas in language-eloquent areas

Study	*N*	Tumour location, *n* (%)	Language assessment tool	Early language deficit	Early assessment timepoint	Early deficit type	Late language deficit, *n*/*N* (%)	Late assessment timepoint	Persistent deficit *n*/*N* (%)	Recovery pattern
Chang et al. [13]	19	Frontal lobe 12 (64%), parietal lobe 1 (5%), frontotemporal 3 (16%), frontoparietal 1 (5%), fronto-insula 1 (5%), insula 1 (5%)	sfBDAE, DO80, PPTT	NR	1 week post-op	Decline in sfBDAE	NR	3 months post-op	NR	Intraoperative DO 80/PPTT correlated with 1-week sfBDAE scores but not with 3-month scores. Resection was stopped when naming errors appeared on DO 80 or responses were incorrect on PPTT. 53% WHO grade 3–4; total resection (> 95%) in 11/19 (58%), subtotal in 3/19 (16%)
Kahana et al. [14]	28 patients/32 operations (15 GBM WHO grade 4; 13 WHO grade 2–3)	Frontal 3 (11%), temporal 5 (18%), parietal 8 (29%), insula 5 (18%), SMA 6 (21%), Frontoparietal 1 (4%)	Picture naming, Reading (word/non word), Sentence repetition, Syntax tasks PPTT	NR	Intraoperative	Naming errors, speech arrest, repetition failure, reading errors. Subcortical stimulation failed to evoke language interference in 11/32 (34.4%) cases. Preoperative language deficits were documented per patient (including dyslexia, syntax, naming and phonological buffer deficits), and intraoperative task requirements were adjusted to baseline ability	NR	Not reported (no postoperative language follow-up)	NR	NR
Li et al. [15]	225 (AAA: 41, MAC: 184)	AAA: frontal 14 (34.1%), temporal 3 (7.3%), parietal 8 (19.5%), two or more brain lobes 16 (39.0%) MAC: frontal 54 (29.3%), temporal 25 (13.6%), parietal 18 (9.8%), two or more brain lobes 46 (25%)	Western Aphasia Battery (WAB), Modified Rankin Scale (MRS)	AAA: post op day 1: 6 (14.6%), post-op day 3: 10 (24.4%), post-op day 5: 10 (24.4%), post-op day 7: 6 (14.6%); MAC: post-op day 1: 6 (3.3%), post-op day 3: 14 (7.6%), post-op day 5: 8 (4.3%), post-op day 7: 4 (2.2%)	1, 3, 5, 7 days post-op	Language, motor, sensory	NR	1, 3, 6 months post-op	0(0%)	No persistent language deficits by 90 days
Papagno et al. [16]	44	Frontal and temporal (frontal UF removal *n* = 8; temporal UF removal *n* = 10; no UF removal *n* = 26)	Verbal fluency on phonological and semantic cue, Famous face naming, picture naming of objects and actions, naming by description, pointing to a picture, sentence comprehension, repetition of words and sentences	Famous face naming below cut-off, UF removed 7/18 (39%); UF preserved 3/26 (12%); pooled 10/44 (23%)	3–7 days post-op	Proper name anomia (famous faces), reduced verbal fluency, memory deficits, especially in UF removal groups	Famous face naming below cut-off, UF removed 8/17 (47%); UF preserved 2/20 (10%); pooled 10/37 (27%)	3 months post-op	UF removed 8/17 (47%); UF preserved 2/20 (10%)	Partial recovery; deficits higher after UF removal. Note: 44 enrolled but 37 assessed at 3 months (5 UF-preserved patients not examined; 2 did not complete all tasks), hence differing denominators
Saito et al. (2016) [17]	82 (with PT: 18, without PT: 64)	Frontal (100%) (dominant hemisphere, PT subgroup analysed)	Naming, reading verb generation, spontaneous speech monitoring (evaluation of recall, counting, fluency and comprehension)	4/18 (22%) of the PT group	1 week post-op	Motor aphasia, speech disturbance, paraphasia, repetition error	1/18 (5.6%)	6 months post-op	1/18 (5.6%)	Most early deficits resolved by 6 months
Saito et al. (2022) [18]	7	Frontal (100%) (frontal operculum, middle frontal gyrus, inferior frontal gyrus, precentral gyrus)	Naming, repetition, spontaneous speech	7/7 (100%)	Immediately post-op	Mild paraphasia, naming object disturbance, repetition error	0/7 (0%) at final follow-up (all recovered; 1/7 required 24 months)	0.5–24 months (per-patient time to recovery)	0/7 (0%)—all patients recovered	6/7 recovered within 0.5–1 month; 1/7 recovered at 24 months (the patient with the greatest CCEP decline). 2/7 had mild preoperative speech disturbance. Mean EOR 93% (85–98%)
Sellier et al. [19]	84 (group 1: 63, group 2: 21)	NR	DO80 naming task	Group 1: 33 (52%), group 2: 17 (81%)	Immediate and 3–5 days post-op	Group 1: Language impairment, sensorimotor deficit, articulation disorder; Group 2 (language disorder)	3/81 (3.7%) permanent neurological deficits, none of which were language deficits	3 months and 1 year post-op	0/81 (0%) persistent language deficit (the 3/81, 3.7% permanent deficits at 1 year were sensorimotor or dysaesthetic)	Most deficits resolved by 1 year. 94% able to work at 3 months postoperatively
Suarez-Meade et al. [20]	15	Frontal 15 (100%), Temporal 1 (6.7%), Insular 8 (53.3%)	Tablet-based NeuroMapper (picture naming paradigm)	2/15 (13.3%) intraoperative naming decline (> 5% error rate vs baseline); 14/15 (93.3%) had a preoperative speech deficit	Intraoperative (continuous neuropsychological monitoring)	Mild expressive aphasia, word-finding difficulty, slower auditory naming, and dysarthria	7/15 (46.7%)	2 weeks	7/15 (46.7%)	Recovery noted in all patients at 2 weeks compared with baseline, though 7/15 (46.7%) still had mild residual speech-language deficits. Mean EOR 60.35% ± 29.60
Tsuzuki et al. [21]	18	Frontal, temporal	SLTA	4 (100%) in NMA resection group	Immediate	Apraxia of speech	NR	1–2 months	0(0%)	Recovered within 3 months All four patients returned to work an average of 5.5 months postoperatively
Vassal et al. [22]	10	Frontal 3 (30%), temporal 4 (40%), parietal 2 (20%), fronto-insular 1 (10%)	NIH stroke scale	80%	< 24 h post-op	Mild aphasia (motor, sensorimotor), language deterioration	50%	1 month	0 (0%)	All deficits resolved by 1 month

*Abbreviations*: *sfBDAE* short form Boston Diagnostic Aphasia Examination, *DO80* Deloche 80 naming test, *PPTT* Pyramids and Palm Trees Test, *AAA* asleep-awake-asleep, *MAC* monitored anaesthesia care, *WAB* Western Aphasia Battery, *UF* uncinate fasciculus, *PT* pars triangularis, *NMA* negative motor area, *NR* not reported

### Clinical implications

While AC with DES supports maximal safe resection in language-eloquent glioma surgery, favourable outcomes cannot be attributed to awake mapping alone; tumour characteristics, patient selection and surgical expertise remain important confounders [7, 25]. Surgical planning should therefore remain individualised, incorporating tumour location, white matter tract involvement, operative feasibility and patient-specific factors. Where key language pathways are implicated, subcortical mapping should be considered integral rather than adjunctive, supporting connectomic approaches to glioma surgery [23]. This is illustrated by the higher deficit rates reported following uncinate fasciculus resection (8/17, 47% on famous face naming at 3 months, versus 2/20, 10% with the tract preserved) compared with the lower rates (0–5.6%) observed in most studies using standardised general language assessment at ≥ 3 months [16]. However, tract involvement may also reflect underlying tumour biology rather than acting as an independent determinant of postoperative outcome.

Preoperative counselling should emphasise that transient postoperative language disturbance is common, whereas persistent deficits are comparatively less frequent [26]. Patients should be informed that most early deficits resolve within three months, although this may not apply uniformly across all clinical contexts. Early speech and language therapy should be considered to support functional recovery although evidence for benefit beyond natural recovery remains limited. Generalisability of the current evidence is further restricted by reliance on specialist multidisciplinary expertise within high-volume centres. Standardisation of outcome reporting through validated language assessments and consistent follow-up intervals of at least three months is required to improve comparability across centres, particularly given the documented variability in perioperative language-testing practice between centres [27] and ensure variation in reported outcomes reflects genuine clinical differences rather than inconsistencies in assessment methodology.

### Future directions

Future research should prioritise standardisation of postoperative language outcome reporting, as heterogeneity in assessment tools and follow-up intervals remains a major limitation of the current evidence base. Adoption of validated language tests paired with predefined assessment intervals would improve comparability and strengthen interpretation of outcomes across studies and institutions. Prospective multicentre studies are required to address the predominance of small retrospective single-centre cohorts. These should stratify findings by tumour grade, molecular characteristics and white matter tract involvement to better account for underlying confounding factors. Building on the association between uncinate fasciculus involvement and persistent language deficits identified in this review, further work should evaluate the role of systematic subcortical DES within connectomic approaches to surgical planning, particularly whether routine subcortical mapping improves long-term language outcomes compared with cortical-only strategies. Greater methodological consistency may also facilitate future meta-analytic synthesis, which is currently limited by substantial heterogeneity in outcome reporting.

Longitudinal study designs are required to better characterise recovery trajectories and distinguish transient from persistent postoperative deficits across standardised timepoints. The role of speech and language therapy in recovery remains poorly defined and warrants investigation through prospective randomised designs, particularly regarding whether early intervention improves functional recovery beyond natural resolution. Greater incorporation of patient-reported outcomes and QoL measures is essential to capture the functional impact of postoperative language deficits from the patient perspective. Lastly, as most evidence is derived from specialist high-volume centres, future work exploring centralised models will be important for improving the consistency of outcome reporting and enhancing generalisability across diverse healthcare settings.

## Conclusion

Awake craniotomy with DES continues to be regarded as the gold standard procedure for preserving language function during the resection of diffuse gliomas situated in eloquent language areas. Early postoperative deficits were frequently observed and generally showed improvement over time, with most resolving within three months. Persistent deficits at or beyond three months ranged from 0 to 47%; specifically, 0–5.6% in studies that reported outcomes for the entire cohort using standardised assessment tools, and 47% observed in patients undergoing uncinate fasciculus resection, notably in a single cohort, which was assessed via famous face naming (8/17). Variations in reported early deficit rates were closely linked to assessment timing and methodology; however, genuine differences in outcomes cannot be entirely excluded. Given the pooled data on deficit types, inconsistent preoperative assessments, and variable follow-up durations, these findings should be interpreted as illustrating a broad trend in recovery rather than definitive prognostic indicators. The current evidence does not support a universal intraoperative stopping criterion nor endorse a single optimal assessment tool. Consider systematic subcortical mapping when critical language pathways are involved, and use standardised perioperative assessments with follow-up periods of at least three months. Nonetheless, prospective multicentre studies with consistent reporting protocols are still necessary.

## Supplementary Information

Supplementary Material 1.

Supplementary Material 2.

## Data Availability

No datasets were generated or analysed during the current study.

## Abbreviations

AAA: Asleep-Awake-Asleep
AF: Arcuate fasciculus
CCEP: Cortico-cortical evoked potentials
CENTRAL: Cochrane Central Register of Controlled Trials
DES: Direct electrical stimulation
DO80: Deloche 80 Naming Test
DTI-FT: Diffusion Tensor Imaging Fibre Tracking
EOR: Extent of resection
FAT: Frontal aslant tract
FLA: Frontal language area
fMRI: Functional magnetic resonance imaging
GTR: Gross total resection
IEMAS: Intraoperative examination monitoring for awake Surgery
IFOF: Inferior fronto-occipital fasciculus
ILF: Inferior longitudinal fasciculus
JBI: Joanna Briggs Institute
KPS: Karnofsky Performance Status
MAC: Monitored anaesthesia care
MEP: Motor evoked potential
MMSE: Mini-Mental State Examination
MRS: Modified Rankin Scale
NIHSS: National Institutes of Health Stroke Scale
NMA: Negative motor area
NR: Not reported
PLA: Posterior language area
PPTT: Pyramids and Palm Trees Test
PR: Partial resection
PRISMA: Preferred Reporting Items for Systematic Reviews and Meta-Analyses
PROSPERO: International Prospective Register of Systematic Reviews
PT: Pars triangularis
sfBDAE: Short-form Boston Diagnostic Aphasia Examination
SLF: Superior longitudinal fasciculus
SLTA: Standard Language Test of Aphasia
SMA: Supplementary motor area
STR: Subtotal resection
SubCF: Subcallosal fasciculus
UF: Uncinate fasciculus
vPMC: Ventral premotor cortex
WAB: Western Aphasia Battery
WHO: World Health Organization
