# Supplementary material for: Beyond the cortex, early deficits, late recovery: a systematic review of language outcomes and subcortical mapping after awake craniotomy for diffuse gliomas
Source: Chin Neurosurg J. 2026 Nov 9;12:33. doi: 10.1186/s41016-026-00452-8 (PMC13746491; doi:10.1186/s41016-026-00452-8)
Supplement: Supplementary file 1 [file 41016_2026_452_MOESM2_ESM.docx]

**A search strategy for Postoperative Language Outcomes After Awake Craniotomy with Direct Electrical Stimulation Language Mapping for Diffuse Gliomas in Language-Eloquent Areas: A Systematic Review**

Four databases (PubMed, Embase via ovid, Medline, and Cochrane CENTRAL) will be searched. All references will be imported into Rayyan for deduplication and screening. Title and abstract screening as well as Full-text screening will be done independently by three reviewers. Discrepancies will be resolved through consensus and discussion.

**Pubmed: 192 records obtained on 01/03/2026**

("Glioma"[Mesh] OR "Neurosurgical Procedures"[Mesh] OR "low grade glioma"[Title/Abstract] OR "LGG"[Title/Abstract] OR "diffuse glioma"[Title/Abstract] OR "diffuse astrocytoma"[Title/Abstract] OR "oligodendroglioma"[Title/Abstract] OR "IDH mutant"[Title/Abstract] OR "IDH wildtype"[Title/Abstract] OR "WHO grade 2"[Title/Abstract] OR "WHO grade 3"[Title/Abstract]) AND ("awake"[Title/Abstract] OR "awake craniotomy"[Title/Abstract] OR "awake surgery"[Title/Abstract] OR "intraoperative mapping"[Title/Abstract] OR "cortical stimulation"[Title/Abstract] OR "direct electrical stimulation"[Title/Abstract] OR "DES"[Title/Abstract]) AND ("asleep"[Title/Abstract] OR "general anesthesia"[Title/Abstract] OR "neurophysiological monitoring"[Title/Abstract] OR "intraoperative monitoring"[Title/Abstract] OR "motor evoked potential"[Title/Abstract] OR "MEP"[Title/Abstract] OR "somatosensory evoked potential"[Title/Abstract] OR "SSEP"[Title/Abstract] OR "diffusion tensor imaging"[Title/Abstract] OR "DTI"[Title/Abstract] OR "tractography"[Title/Abstract] OR "functional mapping"[Title/Abstract]) AND ("language"[Title/Abstract] OR "aphasia"[Title/Abstract] OR "speech"[Title/Abstract] OR "naming"[Title/Abstract] OR "fluency"[Title/Abstract] OR "comprehension"[Title/Abstract] OR "dominant hemisphere"[Title/Abstract] OR "left hemisphere"[Title/Abstract] OR "Broca"[Title/Abstract] OR "Wernicke"[Title/Abstract] OR "eloquent area"[Title/Abstract] OR "eloquent cortex"[Title/Abstract]) NOT ("glioblastoma"[Title/Abstract] OR "GBM"[Title/Abstract] OR "gliosarcoma"[Title/Abstract] OR "Grade 4"[Title/Abstract] OR "high grade"[Title/Abstract] OR "HGG"[Title/Abstract] OR "animal"[Title/Abstract] OR "animals"[Title/Abstract] OR "rat"[Title/Abstract] OR "rats"[Title/Abstract] OR "mouse"[Title/Abstract] OR "mice"[Title/Abstract])

**Medline via ovid: 40 records obtained on 01/03/2026**

(exp Glioma/ OR exp Neurosurgical Procedures/ OR (low grade adj2 glioma).ti,ab,kw. OR LGG.ti,ab,kw. OR (diffuse adj2 (glioma OR astrocytoma)).ti,ab,kw. OR oligodendroglioma.ti,ab,kw. OR (IDH adj2 (mutant OR wildtype OR wt)).ti,ab,kw. OR (WHO adj2 (grade 2 OR grade 3 OR grade II OR grade III)).ti,ab,kw.) AND (exp Consciousness/ OR exp Intraoperative Period/ OR awake.ti,ab,kw. OR (awake adj2 craniotomy).ti,ab,kw. OR (awake adj2 surgery).ti,ab,kw. OR (intraoperative adj2 mapping).ti,ab,kw. OR (cortical adj2 stimulation).ti,ab,kw. OR (direct adj2 electrical adj2 stimulation).ti,ab,kw. OR DES.ti,ab,kw.) AND (asleep.ti,ab,kw. OR (general adj2 anesthesia).ti,ab,kw. OR (neurophysiological adj2 monitoring).ti,ab,kw. OR (intraoperative adj2 monitoring).ti,ab,kw. OR (motor adj2 evoked adj2 potential*).ti,ab,kw. OR MEP.ti,ab,kw. OR (somatosensory adj2 evoked adj2 potential*).ti,ab,kw. OR SSEP.ti,ab,kw. OR (diffusion tensor adj2 imaging).ti,ab,kw. OR DTI.ti,ab,kw. OR tractography.ti,ab,kw. OR (functional adj2 mapping).ti,ab,kw.) AND (language.ti,ab,kw. OR aphasia.ti,ab,kw. OR speech.ti,ab,kw. OR naming.ti,ab,kw. OR fluency.ti,ab,kw. OR comprehension.ti,ab,kw. OR (dominant adj2 hemisphere).ti,ab,kw. OR (left adj2 hemisphere).ti,ab,kw. OR Broca.ti,ab,kw. OR Wernicke.ti,ab,kw. OR (eloquent adj2 (area* OR cortex)).ti,ab,kw.) NOT (glioblastoma.ti,ab,kw. OR GBM.ti,ab,kw. OR gliosarcoma.ti,ab,kw. OR (Grade 4 OR high grade OR HGG).ti,ab,kw. OR animal*.ti,ab,kw. OR rat.ti,ab,kw. OR rats.ti,ab,kw. OR mouse.ti,ab,kw. OR mice.ti,ab,kw.)

**Embase (Ovid): 222 records obtained on 01/03/2026**

(exp low grade glioma/ OR exp diffuse glioma/ OR exp astrocytoma/ OR exp oligodendroglioma/ OR (low grade adj2 glioma).ti,ab,kw. OR LGG.ti,ab,kw. OR (diffuse adj2 (glioma OR astrocytoma)).ti,ab,kw. OR oligodendroglioma.ti,ab,kw. OR (IDH adj2 (mutant OR wildtype OR wt)).ti,ab,kw. OR (WHO adj2 (grade 2 OR grade 3 OR grade II OR grade III)).ti,ab,kw.) AND (exp wakefulness/ OR exp intraoperative period/ OR awake.ti,ab,kw. OR (awake adj2 craniotomy).ti,ab,kw. OR (awake adj2 surgery).ti,ab,kw. OR (intraoperative adj2 mapping).ti,ab,kw. OR (cortical adj2 stimulation).ti,ab,kw. OR (direct adj2 electrical adj2 stimulation).ti,ab,kw. OR DES.ti,ab,kw.) AND (asleep.ti,ab,kw. OR (general adj2 anesthesia).ti,ab,kw. OR (neurophysiological adj2 monitoring).ti,ab,kw. OR (intraoperative adj2 monitoring).ti,ab,kw. OR (motor adj2 evoked adj2 potential*).ti,ab,kw. OR MEP.ti,ab,kw. OR (somatosensory adj2 evoked adj2 potential*).ti,ab,kw. OR SSEP.ti,ab,kw. OR (diffusion tensor adj2 imaging).ti,ab,kw. OR DTI.ti,ab,kw. OR tractography.ti,ab,kw. OR (functional adj2 mapping).ti,ab,kw.) AND (language.ti,ab,kw. OR aphasia.ti,ab,kw. OR speech.ti,ab,kw. OR naming.ti,ab,kw. OR fluency.ti,ab,kw. OR comprehension.ti,ab,kw. OR (dominant adj2 hemisphere).ti,ab,kw. OR (left adj2 hemisphere).ti,ab,kw. OR Broca.ti,ab,kw. OR Wernicke.ti,ab,kw. OR (eloquent adj2 (area* OR cortex)).ti,ab,kw.) NOT (glioblastoma.ti,ab,kw. OR GBM.ti,ab,kw. OR gliosarcoma.ti,ab,kw. OR (Grade 4 OR high grade OR HGG).ti,ab,kw. OR animal*.ti,ab,kw. OR rat.ti,ab,kw. OR rats.ti,ab,kw. OR mouse.ti,ab,kw. OR mice.ti,ab,kw.)

**Cochrane CENTRAL: 9 records obtained on 01/03/2026**

("Glioma" OR "Neurosurgical Procedures") AND ("awake" OR "Consciousness") AND ("general anesthesia" OR "Monitoring, Intraoperative") AND (randomized OR randomised OR controlled OR trial)

Limits: English language and human studies with papers from 2010.
